# Association between infection early in life and mental disorders among youth in the community: a cross-sectional study

**DOI:** 10.1186/1471-2458-11-878

**Published:** 2011-11-21

**Authors:** Renee D Goodwin

**Affiliations:** 1 Department of Epidemiology, Mailman School of Public Health, Columbia University, 722 West 168th Street, Rm 1505, New York, NY 10032, USA

## Abstract

**Background:**

The objective of this study was to examine the association between infection early in life and mental disorders among youth in the community.

**Methods:**

Data were drawn from the MECA (Methods in Epidemiology of Child and Adolescent psychopathology), a community-based study of 1,285 youth in the United States conducted in 1992. Multiple logistic regression analyses were used to investigate the association between parent/caregiver-reported infection early in life and DSM/DISC diagnoses of mental disorders at ages 9-17.

**Results:**

Infection early in life was associated with a significantly increased odds of major depression (OR = 3.9), social phobia (OR = 5.8), overanxious disorder (OR = 6.1), panic disorder (OR = 12.1), and oppositional defiant disorder (OR = 3.7).

**Conclusions:**

These findings are consistent with and extend previous results by providing new evidence suggesting a link between infection early in life and increased risk of depression and anxiety disorders among youth. These results should be considered preliminary. Replication of these findings with longitudinal epidemiologic data is needed. Possible mechanisms are discussed.

## Background

In recent years, there is increasing interest in determining linkages between mental health in early life and risk of poor mental health in adolescence and adulthood [1-5]. Literature to date generally supports a relationship between mental health and early behavioral tendencies and mental health status and psychiatric outcomes later in life. More recently, there is growing interest in extending this link by investigating whether and to what degree physical health in early life is predictive of later mental health and functional outcomes [5,6]. In particular, there has been growing interest in the linkages between early immune functioning and later health outcomes [7-9].

Previous clinical and epidemiologic data have demonstrated links between early immune functioning and later physical health outcomes [10-12]. In general, severe infection has been shown to be associated with increased risk of physical health problems in adolescence and later adulthood. For instance, youth with early respiratory infections have increased risk of respiratory disease (e.g., asthma) in later life [13-15]. Yet, this is not the outcome in all cases, and severity of early insult/illness may play a significant role in this determination.

More recently, there are converging data from several sources suggesting that infection early in life may also be related to poor mental health outcomes. For instance, data from adult cross-sectional epidemiologic samples showing higher rates of schizophrenia and other psychotic disorders among those exposed to early infection [16-18]. More recently, this link has been extended to anxiety disorders among youth [19-23]. Specifically, there is increasing evidence of a relationship between infection early in life and increased likelihood of anxiety disorders later in childhood and adulthood. This association is supported by evidence from two sources. First, there are data to suggest an association between streptococcal infection and increased risk of obsessive-compulsive disorder (OCD) in clinical samples [20-23]. Second, data from community-based samples suggest that there is a relationship between early respiratory disease and increased risk of panic-related disorders during young adulthood [19].

There are also convergent data from laboratory studies suggesting that infection may lead to changes in immune function (e.g., shift from T1 to T2 immune functioning) and that these may relate to increased depressive symptoms and behaviors [24-26].

In sum, there is growing interest and evidence regarding an association between infection early in life and increased risk of later poor mental health outcomes. Yet, several questions remain unanswered. First, it is not known whether there is an association between early infection and increased risk of any mental disorders, or if the risk specific to only some disorders. Second, it is not clear whether the association between early infection and later increased risk of mental disorders applies to the community, or whether it is only evident in selected clinical samples.

Against this background, the goal of the current study is to determine the association between infection early in life and the risk of mental disorders among youth in the community. The study will: (1) investigate the association between early infection and the odds of any mental disorders in youth, compared to youth without early infection; (2) investigate the association between early infection and specific mental disorders in youth, compared with those without early infection.

## Methods

### Sample

Data were drawn from the Methods in Epidemiology of Child and Adolescent psychopathology (MECA) study, a cross-sectional epidemiologic study designed to gather data on childhood psychopathology with structured clinical interviews of both the child and parent (U01MH46718) [27,28]. The MECA study was conducted at 4 sites in the United States in which 1,285 youth were surveyed: Columbia University, Emory University, the University of Puerto Rico, and Yale University.

### Measures

In the MECA Study, one child and one parent/guardian in each family were interviewed and detailed information on the child was obtained, including child psychiatric diagnoses (measured by NIMH's DISC 2.3, including major depression, dysthymia, separation anxiety disorder, panic disorder, social phobia, specific phobia, overanxious disorder, avoidant disorder, attention deficit hyperactivity disorder, conduct disorder, oppositional defiant disorder and substance use disorders). Further details of the MECA methods are available elsewhere [29,30].

### Early infection

A question as asked to all parents/caregivers of the respondent whether the child had experienced, "a severe infection during the first year of life, needing antibiotics." Responses were yes/no.

### Analytic strategy

First, Pearson's Chi-Square tests were used to examine differences in age, sex and socioeconomic status between youth with and without early infection. All tests were two-tailed and significance was set at .05. Next, multiple logistic regression analyses were conducted to determine the relationship between early infection and mental disorders. In conducting these analyses, unadjusted odds ratios were computed, with 95% confidence intervals.

### Approval

This research project was in compliance with the Helsinki Declaration. Columbia University Institutional Review Board granted ethical approval of this study.

## Results

There were no statistically significant differences between youth with and without early infection in terms of age (12.9 (2.2) vs. 12.9 (2.6) years, p = .96), sex (57.1% vs. 53.0% female, p = .8) or low socioeconomic status (14.3% vs. 15.0%, p = .9).

Table 1 shows the results of bivariate associations between early infection and mental disorders during childhood. Major depression, panic disorder, social phobia, overanxious disorder and oppositional defiant disorder (ODD) were significantly more common among youth with a history of infection, compared to youth without infection.

**Table 1:** Association between infection early in life and mental disorders among youth in the community

	No infection (n = 1265)	Infection (n = 14)	OR* (95% CI)
**Any mental disorder**	31.8% (404)	64.3% (9)	**3.9** **(1.3, 11.6)**
Major depression	6.9% (88)	21.4% (3)	**3.7** **(1.0, 13.4)**
Dysthymia	4.3% (55)	0	n/a
Separation anxiety disorder	6.5% (83)	7.1% (1)	1.1 (0.1, 8.5)
Panic disorder	0.6% (8)	7.1% (1)	**12.1** **(1.4, 104.2)**
Social phobia	14.7% (187)	50.0% (7)	**5.8** **(2.0, 16.7)**
Overanxious disorder	11.0% (140)	42.9% (6)	**6.1** **(2.1, 17.7)**
Obsessive or compulsive disorder	2.8% (35)	7.1% (1)	2.7 (0.3, 21.3)
Generalized anxiety disorder	4.6% (59)	14.3% (2)	3.4 (0.7, 15.6)
Avoidant disorder	4.6% (59)	14.3% (2)	3.4 (0.7, 15.6)
Specific phobia	21.6% (275)	21.4% (3)	1.0 (0.3, 3.6)
Attention deficit/hyperactivity Disorder	6.4% (81)	14.3% (2)	2.4 (0.5, 11.1)
Conduct disorder	5.8% (74)	0	n/a
Oppositional defiant disorder	6.9% (88)	21.4% (3)	**3.7** **(1.0, 13.4)**
Any substance use disorder	2.2% (28)	0n/a	

Bold = p < .05

*ORs are unadjusted

Among youth with early infection, 35.7% (5) met criteria for no mental disorder diagnosis, 7.1% (1) had one, 21.4% (3) had two, 7.1% (1) had four, 7.1% (1) had five, 14.3% (2) had six and 7.1% (1) met criteria for eight diagnoses.

## Discussion

These results suggest a possible link between early infection and increased odds of mental disorders among youth in the community. Specifically, the data suggest that early severe infection may be related to increased likelihood of major depression, overanxious disorder, separation anxiety, and specific phobia, compared to those without infection, who were not exposed to infection early in life. Adjustment for confounding factors suggested that this link was not attributable to differences in sociodemographic characteristics.

These data suggest that there is an association between severe infection early in life and increased risk of depressive and anxiety disorders during childhood and adolescence. The mechanism of this association cannot be determined on the basis of these data alone, though three main possibilities seem plausible. It may be that experience of a severe infection leads to functional impairment or limitations later in life such that demoralization and depression are more likely to result. For instance, several studies have shown linkages between physical illnesses and post-traumatic stress disorder [31] and depressive disorders [32]. It is also possible that early infection leads to biochemical changes which result in neurobiological differences that increase the risk of depression onset. This explanation is consistent with laboratory findings showing linkages between immune functioning and depressive behaviors [24-26]. It is also consistent with literature on sickness behavior and depressive symptoms [33,34]. Alternatively, it may be that there are common factors that lead to increased likelihood of having both infection in early life and mental disorders during childhood. These include parental psychopathology or potentially adverse childhood exposures such as childhood abuse or childhood neglect both of which are associated with mental disorders in childhood,[35,36] though less is known specifically about linkages between childhood abuse and infection early in life. Finally, it is also possible that severe infection during youth lead to increased anxiety disorders among vulnerable parents and that this correspondingly increased the risk of anxiety and depressive disorders among youth, as parental and offspring internalizing disorders are linked [37].

### Study limitations

There are several limitations of this study that need to be considered when interpreting results, which should be viewed as preliminary until replication. First, the measure of early infection was very crude, as it was by parent report only and therefore could be influenced by recall or misinformation bias. As reporting was retrospective, failure to recall early infection is possible, and it is conceivable that parents of youth with more serious mental disorders may be more likely to recall problems such as illness during early years of life. Ideally, a future study that uses prospective data with corroborative information from physical examination, laboratory tests, medical records, or data on type of treatment received can replicate these findings. Also, use of a larger sample to test this hypothesis is recommended as cell size is small in several analyses and may have limited our ability to detect statistical significance in some comparisons.

## Conclusions

Results of this study provide preliminary results suggesting early severe infection in childhood is associated with an increased risk of depressive and anxiety disorders during childhood and adolescence. Future studies with more rigorous measurement of infection are needed for replication of these results and to understand potential mechanisms of the observed link.

## Abbreviations

MECA: Methods in Epidemiology of Child and Adolescent psychopathology; DSM: Diagnostic Statistical Manual; DISC: Diagnostic Interview Schedule for Children; OR: odds ratio; OCD: obsessive-compulsive disorder; NIMH: National Institute of Mental Health; ODD: oppositional defiant disorder.

## Competing interests

The authors declare that they have no competing interests.

## Authors' contributions

RG conceived of the study, created its design, wrote the manuscript, and performed the statistical analysis.

## Pre-publication history

The pre-publication history for this paper can be accessed here:

http://www.biomedcentral.com/1471-2458/11/878/prepub
